# Atypical Inter-Brain Synchrony and Social Communication Deficits in Girls with Fragile X Syndrome: Evidence from Functional Near-infrared Spectroscopy Hyperscanning

**DOI:** 10.21203/rs.3.rs-7541575/v1

**Published:** 2025-10-17

**Authors:** Rihui Li, Henrikke Dybvik, Danyong Feng, Cassie Eng, Cindy H. Lee, Kristi L. Bartholomay, Yingchun Zhang, Amy A. Lightbody, Allan L. Reiss

**Affiliations:** University of Macau; Stanford University; University of Macau; Stanford University; Stanford University; Stanford University; University of Miami; Stanford University; Stanford University

**Keywords:** fragile X syndrome, inter-brain synchrony, fNIRS hyperscanning, social communication, Linguistic dysfunction, Autistic symptom

## Abstract

**Background:**

Fragile X syndrome (FXS), a leading genetic cause of intellectual disability and autism, is characterized by marked social communication deficits, yet the neural underpinnings of these challenges remain poorly understood.

**Methods:**

We employed a functional near-infrared spectroscopy(fNIRS) hyperscanning technique to investigate inter-brain synchrony (IBS) during naturalistic mother-child interactions in girls with FXS (n = 33), compared to age- and verbal IQ-matched controls (n=18)and typically developing (TD) peers (n=12). Dyads engaged in a cooperative tangram task and a free-talk conversation, while fNIRS measured neural activity across prefrontal, temporal, and parietal regions.

**Results:**

During a tangram task, the FXS dyads showed distinct IBS patterns, including significantly enhanced IBS in the frontopolar area and reduced IBS in left Broca’s area and dorsolateral prefrontal cortex. In the conversation, FXS dyads showed significantly reduced IBS in the frontal eye field and superior temporal gyrus but enhanced IBS in the right supramarginal gyrus (SMG), a region linked to phonological processing. Linguistically, children with FXS demonstrated significantly lower lexical richness and syntactic complexity compared to their peers. Stronger IBS in the right SMG correlated with better verbal performance (*r* = 0.267~0.401, *p* < 0.05) and higher autism severity (*r* = 0.276, *p* = 0.035).

**Conclusions:**

By integrating neural and linguistic metrics, our study pioneers an effective framework for FXS social dysfunction, underscoring IBS as a potential biomarker for FXS and informing targeted interventions leveraging dyadic synchrony to enhance communication outcomes.

## Introduction

Fragile X syndrome (FXS), the most common inherited cause of intellectual disability and autism spectrum disorder (ASD) (1), arises from a mutation in the *FMR1* gene that silences the production of the fragile X messenger ribonucleoprotein (FMRP) (2). This neurodevelopmental disorder is characterized by a constellation of cognitive, social, and linguistic deficits, including impaired executive functioning, social anxiety, gaze avoidance, and dysfluent speech patterns (3, 4). To date, up to 60% of individuals with FXS meet diagnostic criteria for ASD (5), underscoring the overlap in social communication challenges between the two conditions. Despite advances in understanding the genetic and molecular underpinnings of FXS, the neural mechanisms linking *FMR1* dysfunction to real-world social and communicative impairments remain poorly elucidated. This gap hinders the development of targeted interventions to address the core deficits in FXS.

A growing body of neuroimaging studies highlights the role of inter-brain synchrony (IBS), a direct measurement of brain-to-brain coupling between interacting individuals, as a critical mechanism for successful social interaction (6–8). IBS has been implicated in joint attention (9), empathy (10), and cooperative tasks (8, 11), with hyperscanning techniques such as functional near-infrared spectroscopy (fNIRS) revealing synchronized activation in key brain regions of dyads such as the dorsal lateral prefrontal (DLPFC) and temporoparietal junction (TPJ) during social interaction (12, 13). In neurotypical populations, stronger IBS correlates with better collaboration, communication fluency, and task performance (14). Conversely, preliminary evidence has reported atypical IBS in children with ASD (15), suggesting its potential as a biomarker for social dysfunction. However, no studies to date have investigated IBS in FXS, despite its high prevalence of social and linguistic impairments. This omission is striking, given that FXS offers a genetically homogeneous model to explore how specific neurobiological aberrations (e.g., synaptic dysregulation due to FMRP absence) translate into aberrant social-brain dynamics.

FXS is also marked by pronounced communication deficits, including reduced syntactic complexity, lexical diversity, and conversational reciprocity (16, 17). These challenges are often compounded by comorbid anxiety and social avoidance, further limiting opportunities for meaningful interaction. While behavioral studies have documented these impairments, their neural correlates, particularly in the context of live social exchanges, remain underexplored. Traditional neuroimaging approaches (e.g., functional Magnetic Resonance Imaging) face limitations in capturing dynamic, naturalistic interactions, whereas fNIRS hyperscanning provides a unique opportunity to measure IBS during ecologically valid tasks (18–20). By examining how mother-child dyads with FXS coordinate their neural activity during cooperative and conversational tasks, we can gain insights into the neural substrates of their communicative difficulties and identify potential targets for intervention.

In this study, we aimed to uncover the neural mechanism of social deficits and linguistic communication patterns in children with FXS during real-time social interaction. A critical innovation of this study lies in its exclusive focus on girls with FXS, a population often underrepresented in previous FXS or ASD research (3, 21, 22). Females with FXS typically exhibit milder cognitive and behavioral phenotypes compared to males, owing to X-chromosome inactivation patterns that partially preserve FMRP expression (23). However, they remain at high risk for social communication deficits, anxiety, and adaptive functioning challenges. By concentrating on female participants, our study aimed to minimize effects associated with lower cognitive function and provide a clearer window into FXS-specific neural and behavioral profiles. To further isolate syndrome-related abnormalities, we included two control groups, a verbal IQ- and age-matched control group of girls without FXS, and a typically developing (TD) group of age-matched girls with no developmental concerns. This design allows us to potentially disentangle deficits intrinsic to FXS from those attributable to general language delays or neurotypical variation. Two interactive tasks were employed in the present study, including a cooperative tangram puzzle, requiring joint problem-solving, and a free-talk conversation, eliciting spontaneous verbal exchange. We hypothesized that FXS dyads would demonstrate atypical IBS patterns during social interactions compared to language- (i.e., verbal IQ) and age-matched controls and TD peers, reflecting their documented difficulties in social interaction. Furthermore, we predicted that atypical IBS would correlate with both linguistic performance (e.g., mean length of utterance) and autism symptom severity, providing a neural basis for the behavioral phenotype of FXS.

## Method and materials

### Participants

We recruited a total of 63 child-mother dyads in this study, including 33 FXS dyads (girls with FXS), 18 control dyads (girls matched for age and verbal IQ), and 12 typically developing (TD) dyads (healthy children matched for age). A diagnosis of FXS (full mutation) was confirmed by molecular genetic testing. Girls in the control and TD groups were enrolled through parent organizations, regional centers, school districts, social media, and flyer distribution. We conducted the research following the Declaration of Helsinki, approved by the Stanford University Institutional Review Board. Participants and their parents/guardians were fully informed about the purpose of the research and provided written informed consent/assent before the experiment started.

### Cognitive-behavior assessments

A series of cognitive-behavior assessments were conducted with the participants, including assessment of verbal IQ using the Differential Ability Scales, Second Edition (DAS-II) (24), executive function using Behavior Rating Inventory of Executive Function-Global Executive Composite, Second Edition (BRIEF-2-GEC) (25), and autism symptoms/social behavior based on parent ratings from the Social Responsiveness Scale-2 (SRS-2) (26). We also assessed the participants’ depression, anxiety, and avoidance symptoms using the Anxiety, Depression, and Mood Scale (ADAMS) (ADAMS-General Anxiety/Depressed Mood/Social Avoidance) (27). All behavioral assessments were administered and scored based on standardized procedures according to each respective testing manual. All behavioral assessments, except the DAS-II, were completed by parent/caregiver. The DAS-II score was generated from direct testing of the participant.

### Social interaction tasks

**Figure 1:**
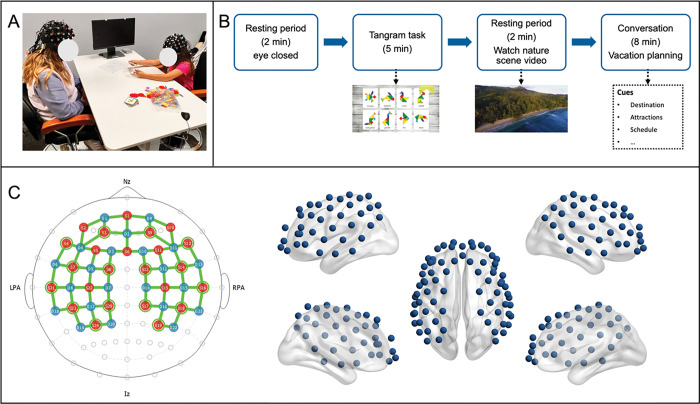
Overview of the experimental design. (A) An example of a child-mother dyad; (B) The experimental paradigm including a tangram task and a free-conversation task; (C) The distribution of fNIRS optodes and cortical mapping of all fNIRS channels.

All child-mother dyads participated in two face-to-face interactive tasks in a naturalistic environment, including a tangram task and a free-talk conversation (Fig. 1A–B). Before the beginning of the task, the participants received a comprehensive briefing on the rules and procedures, with a specific emphasis on maintaining active interaction throughout the experiment. The tangram task began with a resting period, in which the participants were instructed to close their eyes and rest for 2 minutes without any communication. Subsequently, participants engaged in a 6-minute cooperative tangram puzzle task, starting from simple to complex puzzles. Following the tangram task completion, participants underwent a 2-minute nature video viewing session to facilitate cognitive reset and mitigate task carryover effects. The conversation task started after the video-watching session, in which they were instructed to discuss a plan for an upcoming vacation. A list of questions was provided to the mother to facilitate the conversation. All sessions were videotaped to enable the coding of communication patterns and behaviors.

### fNIRS data acquisition and preprocessing

We utilized two NIRSport2 systems (NIRx, Berlin, Germany), each with 24 sources, 23 detectors, and 16 short separation detectors to cover bilateral prefrontal, parietal, and temporal cortices of the dyads (Fig. 1C). The near-infrared light was measured at the wavelengths of 760 and 850 mm and the sampling frequency was 7.81 Hz.

The fNIRS data were analyzed using the NIRS Brain AnalyzIR Toolbox (28). First, channels exhibiting low signal quality, characterized by raw data coefficient of variation (c.v.) exceeding 0.1, were excluded from the processing pipeline. Subsequently, raw fNIRS data were converted to optical density and corrected for motion artifacts using the Temporal Derivative Distribution Repair (TDDR) and wavelet filtering approaches. The scalp coupling index (SCI) metric was computed and assessed per channel to further exclude channels with poor signal quality at a threshold of 0.8 (29). The cleaned optical density data were then converted to HbO and HbR concentration changes through the Modified Beer Lamber Law. Finally, short-separation channels were used to regress out the superficial artifacts (e.g., scalp blood flow) in the HbO and HbR signals.

### Inter-brain Synchrony

The IBS between each dyadic channel pair was calculated using wavelet transform coherence (WTC), a traditional approach widely used in fNIRS-based hyperscanning to assess brain-to-brain coupling (11, 30). For each channel pair, WTC matrixes (frequency Í time) were computed and averaged across time for each task period. Similar to previous studies (31, 32), we employed a permutation test by running a series of independent t-tests between the WTC of each frequency bin of the real samples and that of 1000 randomly generated samples, from which the significant task-related frequency bins can be identified. We then averaged all the identified frequency bins, resulting in a Fisher’s z transformed-WTC value as IBS strength for each channel pair of the dyads. Note that the IBS was calculated for each interactive task separately.

### Communication pattern coding

We employed CLAN (Computerized Language ANalysis, https://dali.talkbank.org/clan/) software to code the communication patterns during the mother-child conversation. First, the audio track of the dyadic conversation was transcribed into text using OpenAI’s Whisper model (https://github.com/openai/whisper), followed by a manual correction to ensure the precision of the transcription. The transcripts were then annotated to distinguish speaker turns and utterance boundaries. Subsequently, CLAN’s computational modules (sugar, mlt, kideval) were applied to extract 4 predefined conversation metrics for each child, including Mean Length of Utterance in Morphemes (MLU), Total Number of Words (TNW), Words per Sentence (WPS), and Clauses per Sentence (CPS). These metrics were selected to evaluate the child participant’s lexical richness, grammatical sophistication, and conversational fluency.

### Statistical analysis

For IBS during the tangram task, one-way analysis of variance (ANOVA) and post-hoc t-tests (two-tailed) were used to assess whether there were significant differences in the IBS and tangram score between the dyads with FXS and the other two groups. For IBS during the conversation task, one-way ANOVA and post-hoc t-tests (two-tailed) were used to assess whether there were significant differences in the IBS and coded communication patterns between the FXS and the other two groups. Multiple comparisons were corrected by false discovery rate using the Benjamin-Hochberg approach at a significant level of 0.05.

For the significant channels identified with atypical task-related IBS, we used Kendall’s correlation to examine the relation between the IBS and social dysfunction (i.e., SRS-2) of the children with FXS. We also examined Kendall’s correlation between the task-related behaviors (i.e., tangram score and communication patterns), the IBS, and the social dysfunction of the children with FXS.

## Results

### Participants demographics

Participant characteristics are summarized in Table 1. The three groups did not differ significantly for age (*p* = 0.589). Significant differences between the FXS and control group were not observed in multiple cognitive-behavior assessments, including verbal IQ (*p* = 0.098) and non-verbal IQ (*p* = 0.056), executive functioning (*p* = 0.129), autism-related social behavior (SRS-2 Total score, *p* = 0.343; SRS-2 RIRB, *p* = 0.209), general anxiety (*p* = 0.422), and social avoidance (*p* = 0.334).

Regarding the tangram task performance, there was a significant difference between the FXS and TD groups (*p* = 0.015), while there was no significant difference between the control and FXS groups or the TD groups.

### Inter-brain synchrony during the tangram task

**Figure 2:**
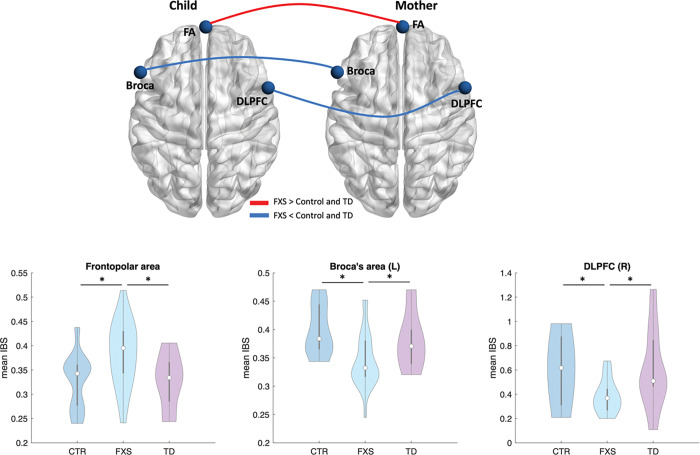
The inter-brain synchrony (IBS) between all three groups during the tangram task. “*” indicate significant difference (*p* < 0.05).

The one-way ANOVA and post-hoc pair-wise analysis revealed a series of significant differences in IBS between the FXS dyads and control or the TD dyads during the two interactive tasks (Fig. 2). When engaging in the tangram task, child-parent dyads in the FXS group showed significantly stronger IBS in the right frontopolar area (FA) compared to the control and TD groups (*p*_*FXS*>*Control*_ = 0.019, *p*_*FXS*>*TD*_ = 0.034). On the contrary, the FXS group revealed significantly weaker IBS in the left Broca’s area (*p*_*FXS*<*Control*_ = 0.001, *p*_*FXS*<*T*D_ = 0.049) and the right dorsal lateral prefrontal cortex (DLPFC, *p*_*FXS*<*Control*_ = 0.010, *p*_*FXS*<*TD*_ = 0.021) compared to the control and TD groups.

### Inter-brain synchrony during the conversation task

**Figure 3:**
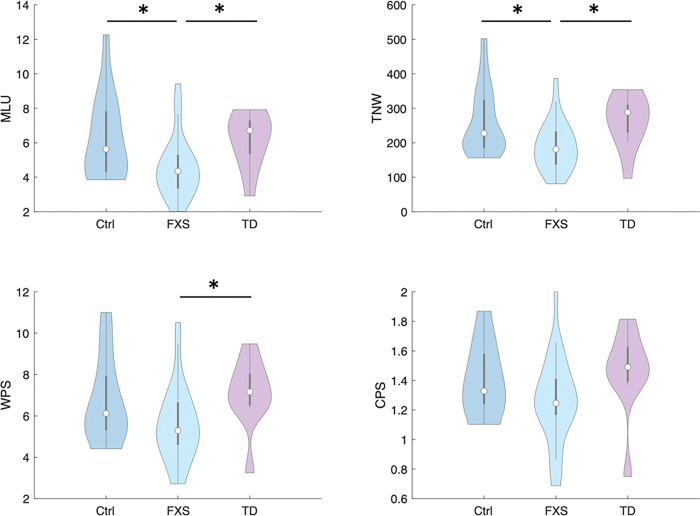
The inter-brain synchrony (IBS) between all three groups during the conversation task. “*” indicate significant difference (*p* < 0.05).

During the conversation, the FXS group showed significantly weaker IBS in the left SFG (*p*_*FXS*<*Control*_ = 0.044, *p*_*FXS*<*TD*_ = 0.0021), left STG (*p*_*FXS*<*Control*_ = 0.021, *p*_*FXS*<*TD*_ = 0.038), and the left FEF (*p*_*FXS*<*Control*_ = 0.007, *p*_*FXS*<*TD*_ = 0.004) compared to the control and TD groups. In addition, the FXS group showed significantly enhanced IBS at the right SMG of Wernick’s area (*p*_*FXS*>*Control*_ = 0.031, *p*_*FXS*>*TD*_ = 0.005) compared to the control and TD groups (Fig. 3).

### Altered communication patterns in FXS dyads

The measured verbal ability of all children during the conversation task, as evidenced by coded communication patterns, is summarized in Table 2. Compared to the control and TD peers, children with FXS demonstrated altered communication patterns during the conversation with their parents. Specifically, the FXS child-parent dyads showed significantly reduced Mean Length of Utterance (MLU) in Morphemes (*p* = 0.021), total number of words (TNW, *p* = 0.008), and words per sentence (WPS, *p* = 0.028) during the conversation compared to the TD dyads. Similar patterns were also identified in comparisons between the FXS and Control groups, where the FXS dyads showed significantly reduced MLU (*p* = 0.012) and TNW (*p* = 0.007) relative to the control group. No significant differences in coded communication patterns were identified between the control and the TD groups.

### Association between IBS, communication patterns, and autistic symptoms

The association between the IBS and coded communication measures in children with FXS was examined using Kendall’s correlation analysis. There was a significant positive correlation between IBS at the right SMG and each communication metric, including the MLU (*r* = 0.267, *p* = 0.040), TNW (*r* = 0.333, *p* = 0.009), WPS (*r* = 0.401, *p* = 0.002), and CPS (*r* = 0.335, *p* = 0.010). That is, the children with FXS who had stronger IBS at the right SMG area showed better verbal performance with their parents when engaging in the conversation.

We further assessed the association between dyadic IBS patterns and autistic symptoms in children with FXS. There was a significant positive correlation between IBS at the right SMG and SRS-2-total score (*r* = 0.276, *p* = 0.035), indicating that higher autism severity was associated with enhanced IBS at the right SMG area.

We also examined the association between the communication metrics and autistic symptoms in children with FXS. However, no significant correlation was revealed by Kendall’s correlational analysis.

## Discussion

A core behavioral feature of FXS relates to avoidance and hyperarousal during social communication and interactions (21). Yet, limited effort has been devoted to elucidating the neural mechanism underlying naturalistic social interactions in children with this condition. The present study investigated IBS patterns during mother-child interactions in girls with FXS compared to age- and IQ-matched controls and TD peers. Our findings revealed distinct neurobehavioral profiles in FXS dyads, characterized by atypical IBS across specific brain regions during cooperative and conversational tasks and impaired communication patterns. These findings provide novel insights into the neural and behavioral mechanisms underlying social and linguistic challenges in girls with FXS, while highlighting potential biomarkers for individualized treatment for this genetic disorder.

We observed enhanced IBS in the frontopolar area (FA) of the FXS dyads during the tangram task, a brain region that is directly implicated in executive function, higher-order cognitive and social processes (33, 34). This finding aligns with our previous single-brain fNIRS study that showed children with FXS to have greater activation in this region in response to facial and social gaze stimuli in FA (18, 35), possibly representing deficient modulation of neural responses to continuous social stimuli. For the tangram task in this study, the enhanced IBS in the FA in girls with FXS starkly contrasts with the reduced IBS observed in ASD dyads during joint attention tasks, which may underscore divergent neural substrates of social dysfunction. In particular, while ASD is generally characterized by diminished motivation for social interaction (36), FXS may involve maladaptive hyper-attunement driven by hyperarousal. For instance, girls with FXS often display gaze aversion coupled with elevated physiological arousal (e.g., increased anxiety and dilated pupil size) during face-to-face interaction (21, 37), a phenotype distinct from the more pervasive social disengagement in ASD. The role of FA in higher-order cognitive and social processing could position it as a critical hub where genetic-driven excitatory-inhibitory imbalance translates into exaggerated FA engagement during the cognitive task (38). This mechanism may also explain why FXS dyads achieved comparable tangram task performance to controls despite the observed atypical IBS – hyper-IBS could temporarily offset social anxiety, enabling task completion at the cost of heightened neural “effort”. Our findings highlight a crucial distinction between FXS and ASD in the neurobiology of social communication, which may lead to potentially different intervention strategies for these two neurodevelopmental disorders.

Conversely, reduced IBS in a language-related region (i.e., left Broca’s area) of the FXS dyads suggests disrupted function in a region critical for language production. It is well-documented that Broca’s area is involved in syntactic and semantic processing (39), the weak IBS in Broca’s area may be associated with linguistic deficits observed in children with FXS, as evidenced by the reduced MLU and TNW. Apart from its function traditionally associated with language production, Broca’s area is also increasingly recognized for its role in non-verbal social coordination, including action observation and implicit rule-based planning (40, 41). Reduced IBS in this region may thus reflect impaired integration of multi-modal cues necessary for the tangram task. Our finding extends beyond language deficits, suggesting that Broca’s dysfunction in FXS disrupts broader social-cognitive processes critical for cooperative problem-solving.

During the conversation task, the FXS dyads showed reduced IBS in the left SFG and left STG compared to the control and TD groups, providing a potential correlate for atypical social-brain dynamics in FXS. The SFG and STG are key hubs for social perception and interaction(42). The SFG supports mentalizing and intention understanding through its connections with the default mode network (43), while the STG is involved in imitation, observation of others’ actions, and emotion contagion (44). The diminished IBS in these regions represents a neural signature of reduced conversational reciprocity and impaired perspective-taking observed behaviorally in the FXS dyads, as evidenced by our coded communication patterns.

We also observed reduced IBS in the FEF of the FXS dyads compared to the control and TD groups. The FEF plays an essential role in the control of visual attention and the saccadic eye movements (45). This finding may be associated with less eye-to-eye gaze in girls with FXS while they engaged with their mothers during conversation, further underscoring the cascading effects of FXS-related neural dysregulation on social communication. Surprisingly, our previous study has reported both eye-gaze avoidance and dysregulated activity in the FEF of the girls with FXS during a conversation task (21), which can be attributed to the anxiety and gaze avoidance behaviors induced during face-to-face social interaction (37). The current finding of reduced IBS in FEF of the FXS dyads, from the view of brain-to-brain coupling, solidifies our previous hypothesis by bridging the gap between single-brain irregulated eye gaze control and dysfunctional social outcomes, providing potential biomarkers for the assessment of social difficulty in FXS.

Paradoxically, enhanced IBS in the right SMG of Wernicke’s area emerged as a notable finding. This region, typically associated with phonological processing and semantic memory (46), may serve as a compensatory hub in FXS, facilitating alternative pathways for language comprehension when canonical left-hemisphere networks are disrupted. The positive correlation between right SMG-IBS and verbal performance supports this interpretation, suggesting that enhanced cross-brain coupling in this region partially mitigates linguistic impairments.

The atypical communication patterns (i.e., reduced MLU, TNW, and WPS) in FXS dyads align with prior reports of dysfluent, repetitive speech patterns in FXS (47, 48). These deficits likely stem from interactions among genetic, neuroanatomical, and environmental factors. For instance, *FMR1* gene silencing and reduced FMRP disrupt dendritic spine maturation in language-related cortices, impairing synaptic efficiency (38). Furthermore, the lack of correlation between communication metrics and autism symptoms (e.g., SRS-2/ADOS-2) implies that linguistic challenges in FXS are not solely secondary to autism traits but may arise from syndrome-specific mechanisms. The dissociation between social dysfunction and language performance underscores the need for targeted interventions addressing both domains independently.

The positive association between the IBS in right SMG and autism severity (SRS-2 total score) introduces a provocative paradox that stronger neural coupling in this region correlates with both better language performance and greater social impairment. This dual role may reflect a maladaptive compensatory mechanism rather than purely beneficial plasticity. Several explanations may account for this phenomenon. First, IBS in the right SMG could represent excessive reliance on phonological and non-social semantic processing to compensate for impaired social-pragmatic networks in canonical left-hemisphere language areas (49). Such compensation might facilitate concrete word production (e.g., better MLU/WPS) while failing to support higher-order social communication, a core deficit captured by SRS-2. Alternatively, enhanced IBS of mother-child dyads in this region may indicate disproportionate maternal scaffolding to overcome the child’s social challenges, where increased neural coupling could be caused by greater parental social initiation. This interpretation is supported by studies showing that parents of children with social impairment often adopt more directive interaction styles (50, 51).

Several limitations merit attention in the present study. First, the modest sample size, particularly in the TD group (n = 12), limits generalizability and statistical power to detect subtle group differences. Although the inclusion of TD and IQ-matched control groups enables the identification of more distinct neural signatures of social impairment in FXS, heterogeneity in FXS phenotypes (e.g., comorbid anxiety, sensory sensitivities) may confound the IBS measures and post-analysis. Future studies should incorporate a larger group size and granular phenotyping to disentangle these factors. Second, the cross-sectional design precludes causal inferences about how IBS patterns evolve with development or intervention. Longitudinal studies tracking IBS changes alongside behavioral trajectories could elucidate directional relationships. A further limitation pertains to the complexity of integrating multi-dimensional data. While our study concurrently captured neural (i.e., IBS), linguistic, and behavioral metrics, the current analytical framework primarily examined linear correlations between isolated variables. This approach may oversimplify the dynamic, bidirectional interactions among social-brain dynamics, communication behaviors, and clinical phenotypes in FXS. Advanced modeling techniques, such as structural equation modeling to delineate causal pathways or network analysis to map system-level interdependencies, would be critical to unravel these multilayered relationships.

## Conclusion

In summary, this study delineates unique neurobiological signatures of social interaction in FXS, characterized by both deficient and compensatory IBS patterns. By linking neural synchrony to communication deficits and autism symptoms, our findings advance understanding of FXS pathophysiology and open avenues for mechanistically grounded interventions in the future. While limitations exist, this work underscores the value of hyperscanning approaches in unraveling the dynamic neural substrates of neurodevelopmental disorders. Future research integrating longitudinal, multimodal, and genetically informed designs will be critical to translating these insights into clinical practice.

## Figures and Tables

**Figure 4:**
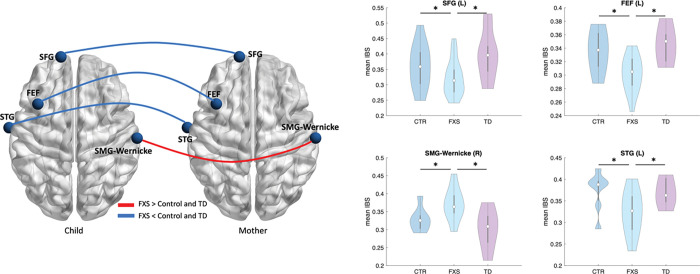
Communication patterns between all groups. “*” indicate significant difference (p < 0.05).

**Table 1:** Characteristics of participants.

Characteristic	Fragile X Syndrome N = 33	Control N = 18	TD N = 12	Statistic
	Mean (SD)	Mean (SD)	Mean (SD)	p value
Age, years	13.2 (2.9)	13.3 (2.9)	12.2 (3.3)	0.589
DAS-II-Verbal^a^	84.0 (18.7)	92.2 (11.5)	/	0.098
DAS-II-Non Verbal^a^	75.6 (20.9)	85.7 (8.3)	/	0.056
BRIEF-2-GEC^b^	56.4 (10.5)	61.8 (11.8)	/	0.129
ADAMS, General Anxiety^c^	5.3 (3.9)	6.3 (4.3)	/	0.422
ADAMS, Social Avoidance^c^	5.1 (3.6)	3.9 (3.9)	/	0.334
SRS-2, Total Score^b^	60.9 (14.0)	65.0 (12.2)	/	0.343
SRS-2, RIRB Score^b^	56.9 (14.7)	62.8 (14.9)	/	0.209
Tangram score	14.2 (7.7)	16.1 (4.7)	21.4 (8.3)	0.015*

a Standard score with mean = 100, SD = 15.

b T scores.

c Raw scores.

**Table 2:** The communication patterns of the children during the conversation task.

Characteristics	FXS	CTR	TD	*p* value		
	Mean (SD)	Mean (SD)	Mean (SD)	FXS vs. CTR	FXS vs. TD	TD vs. CTR
Mean Length of Utterance	4.6 (1.8)	6.3 (2.4)	6.2 (1.6)	**0.012**	**0.021**	0.870
Total Number of Words	188.2 (70.1)	262.9 (99.6)	263.7 (76.1)	**0.007**	**0.008**	0.984
Words Per Sentence	5.6 (1.8)	6.8 (2.0)	7.1 (1.7)	0.052	**0.028**	0.678
Clauses Per Sentence	1.3 (0.3)	1.4 (0.2)	1.5 (0.3)	0.117	0.071	0.549

## Data Availability

The data sets generated and analyzed for this study are not publicly available. Availability of data and code will be considered on reasonable request.
